# New South Wales alcohol and other drug service providers' perceptions of the relative importance of client variables for determining treatment need

**DOI:** 10.1111/dar.13952

**Published:** 2024-10-21

**Authors:** Briony Larance, Isabella Ingram, Chloe Haynes, Lexi Buckfield, Choon Wee Melvin Goh, Peter J. Kelly

**Affiliations:** ^1^ School of Psychology University of Wollongong Wollongong Australia; ^2^ School of Psychology and Public Health La Trobe University Melbourne Australia; ^3^ Centre for Alcohol and Other Drugs New South Wales Ministry of Health Sydney Australia

**Keywords:** addiction, assessment, care planning, routine outcome monitoring, substance use disorder

## Abstract

**Introduction:**

This study examines alcohol and other drug (AOD) service providers' perceptions of the most important variables (client complexity and demographic) for determining treatment need and intensity of intervention.

**Methods:**

Online cross‐sectional survey of *N* = 188 clinicians/service managers working in AOD services across metropolitan and regional/rural New South Wales, Australia. Participants ranked the importance of demographic and family factors, substance use, physical health, mental health, functioning and activities of daily living and youth‐specific variables in identifying treatment need (five‐point Likert scales).

**Results:**

More than 90% of participants ranked 43 out 56 potential variables as ‘very important’/‘essential’ in identifying treatment need. The 10 variables most ranked as ‘very important’/‘essential’ were ‘pregnant or breastfeeding’ (95.2%), ‘suicide/self‐harm’ (95.2%), ‘overdose risk’ (94.7%), ‘abuse/neglect’ (among youth/adolescent populations; 94.1%), ‘mental health severity’ (93.6%), ‘dependent children’ (93.1%), ‘co‐existing mental health concerns’ (93.0%), ‘hospitalisations due to mental health’ (92.5%), ‘child protection concerns’ (among youth/adolescent populations; 92.2%) and ‘disability’ (91.5%). The 10 variables most commonly ranked as ‘slightly important’/‘not at all important’ included ‘citizenship’ (63.3%), ‘sex’ (59.6%), ‘country of birth’ (54.8%), ‘highest education’ (50.0%), ‘sexual orientation’ (44.1%), ‘relationship status’ (33.5%), ‘gender’ (31.4%), ‘transport’ (28.2%), ‘employment’ (23.9%) and ‘refugee status’ (24.0%). Some ratings differed by geographic location (metropolitan vs. regional/rural) and job role (allied health worker, nurse, doctor or manager).

**Discussion and Conclusions:**

This study provides insight into service providers' perceptions of treatment need and intensity associated with a range of client factors. It is a first step towards improvements in routine data collections that are used to inform treatment planning.

**Key Points**

- We examined alcohol and other drug service providers' perceptions of the most important client variables for determining treatment need and intensity of intervention.
- Most service providers ranked most potential variables as ‘very important/essential’ in identifying treatment need, with little differentiation in terms of relative importance.
- The top 10 variables rated as ‘very important/essential’ related to immediate risk of harm to the client (i.e., suicidality/self‐harm, overdose risk), priority populations (i.e., pregnancy or breastfeeding, disability), child protection concerns (i.e., child protection concerns among youth/adolescent populations, dependent children, abuse/neglect among youth/adolescent clients) and mental health comorbidity (i.e., co‐occurrence and severity of mental health concerns, history of hospitalisations due to mental health).
- The 10 variables most rated as ‘slightly important’/‘not at all important’ related to cultural and linguistic diversity (citizenship, country of birth and refugee status), gender identity (sex at birth, gender), sexual orientation, relationship status, socioeconomic status (highest education, employment) and transport issues.
- This study provides insight into service providers' perceptions of treatment need and intensity associated with a range of client factors and is a first step towards improvements in routine data collections.

## 1 INTRODUCTION

People who access alcohol and other drug (AOD) treatment services often present with multiple and complex needs that require consideration in planning and delivering care. This includes, but is not limited to, physical and mental health, homelessness and contact with the criminal justice system (e.g., [1, 2]). In Australia, treatment frequently involves integrating care across service providers, including public sector and health and social service non‐government organisations (NGO) [3, 4]. AOD treatment is also delivered across diverse settings, including inpatient, outpatient, primary care and community locations. While service setting is typically matched to client need, a high degree of diversity exists in the individual interventions provided within a setting. For example, community services might include any combination of opioid agonist treatment, psychosocial interventions, crisis and case management.

In New South Wales (NSW), the Clinical Care Standards describe the key data elements all specialist AOD services collect at intake (e.g., referral reason, AOD use, treatment history, safety assessment, medical and mental health issues, other criteria relevant to priority access), during comprehensive assessment (e.g., reason for presentation, substance use in the past 28 days, substance use in the past 3 days, other AOD treatment, social situation, mental health, physical health, risk relating to client characteristics, circumstances, behaviours and substance use) and throughout treatment (e.g., structured measurements of AOD‐specific issues and general health to assess treatment progress and outcome) [5]. There is some variation in how these standards are operationalised across sectors and services, but enacting them typically involves clinicians collecting data on a wide range of client factors and dimensions. Specialist AOD services in NSW collect and report a standard set of data elements under the NSW Minimum Data Set for Alcohol and other Drug Treatment Services (NSW MDS DATS) [6]. This includes data on client demographics, substance use and service provided. The NSW MDS DATS is reported to the NSW Ministry of Health, who in turn feeds a subset of this data into the Alcohol and Other Drug Treatment Services National Minimum Dataset [7].

Minimum reporting requirements result in routine data collection of some client variables. These data are used for wide ranging purposes by service providers, policy‐makers and funders, including evaluating client outcomes, implementing and supporting care pathways for people experiencing ‘complex’ AOD concerns/issues, facilitating continuous quality improvement projects, supporting internal management and planning systems and developing funding models.

This study is concerned with identifying the subset of client factors, including those not captured by the National Minimum Dataset, that people involved in delivering treatment (including those managing treatment services) consider most important in clinical decision‐making. Specifically, the current study sought to identify the demographic and clinical variables that service providers (service managers and clinicians) believe are most important for capturing/defining the presentation, or ‘treatment need,’ and determining intensity of intervention across different AOD treatment settings. These data will aid our understanding of whether current routinely collected data is consistent with service provider perceptions of what is important. Secondary aims include examining whether perceptions varied according to: (i) geographic setting (metropolitan vs non‐metropolitan); and (ii) job role.

## 2 METHODS

This study draws on data from a cross‐sectional online survey of NSW AOD health service managers and clinicians. No formal sample size calculations were undertaken as the study objectives were descriptive.

### 2.1 Ethics

The study was reviewed and approved by the Joint University of Wollongong and Illawarra Shoalhaven Local Health District Health and Medical Human Research Ethics Committee (ref#: 2021/ETH11327). Relevant site governance approvals were obtained from the following Local Health Districts: Illawarra Shoalhaven, Far West, Mid North Coast, Murrumbidgee, Northern NSW, Southern NSW, Western NSW, Central Coast, Hunter New England, North Sydney, South Eastern Sydney, Sydney, South Western Sydney and Western Sydney, and St Vincent's Hospital Network.

### 2.2 Participants

To be eligible for the study, participants were aged 18 years or older and were currently working in a specialist NSW AOD service. Only those people working in direct client care roles and managers of clinical services were included. Staff who worked in administrative or research roles were excluded.

### 2.3 Procedure

The study was conducted from January 2021 to November 2022. An initial invitation email was distributed to AOD service providers working in government and NGO services across NSW via the Centre for Alcohol and Other Drugs (NSW Ministry of Health) and the Network of Alcohol and Other Drug Agencies (NADA), peak body for NGO AOD services. The email contained a link to the participant information sheet and online survey. Participants initially entered a landing page programmed in Qualtrics XM with a detailed participation information sheet and consent form. Those participants who gave tacit consent continued to commence the survey. The survey was completed anonymously. Participants who failed to meet the eligibility criteria were redirected to a landing page where they were thanked for their time. The total estimated survey completion time was approximately 20 min. Participants were excluded from the final data set for incomplete or unreliable survey attempts.

### 2.4 Measures

The survey collected data on participants' job role, experience and setting in which they worked. The latter sections of the survey asked participants to rank the importance of potential demographic variables in identifying treatment need at entry on a series of five‐point Likert scales (where 1 = ‘not at all important’ and 5 = ‘essential’). This included substance use, physical health, mental health, functioning and youth‐specific variables. Participants were instructed to rate the variables ‘you believe are most important for informing care planning and determining intensity of intervention (note: these variables may differ from the variables that may be important for measuring “treatment experience” or “treatment outcome”)’. The initial pool of client variables included in the survey was drawn from existing routinely collected data collected by NSW treatment service providers, specifically the NSW MDS DATS [8], NSW Non‐Admitted Patient Data Collection [9], the Australian Treatment Outcome Profile (ATOP) [10] and the Client Outcomes Monitoring System (NADABase COMS) [11] (Table A1). This variable list was then reviewed and further refined with input from the investigator team and experts from the Centre for Alcohol and Other Drugs, NSW Ministry of Health and clinician‐researchers, resulting in a final pool of 56 potential variables (Table 1). The order in which participants saw variables for ranking was randomised to minimise order effects and other biases. Only participants who reported working with adolescent or youth populations ranked the youth‐specific variables. Participants were asked to identify other measures or tools they used to make decisions about clients' treatment needs.

**TABLE 1:** Description of labels used in tables and figures (alphabetical order).

Variable label	Domain	Variable description
Adverse life events	Mental health	Exposure to adverse life events (e.g., adverse childhood experiences, trauma, neglect)
ATSI	Demographics	Aboriginal or Torres Strait Islander origin
BBV status	Physical health	Blood borne virus status
Citizenship	Demographics	Citizenship status
CJS involvement	Demographics	Involvement with criminal justice system
Co‐existing mental health concerns	Mental health	Co‐existing mental health concerns
Co‐existing physical health concerns	Physical health	Co‐existing physical health concerns
Cognitive impairment	Functioning	If the person has a cognitive impairment
Country of birth	Demographics	Country of birth
Dependent children	Demographics	If the person is responsible for dependent children
Disability	Functioning	If the person has a disability
DOB	Demographics	Date of birth
Domestic and family violence	Demographics	Experience of domestic or family violence
Employment	Demographics	Employment or work status
Frequency of other substance use	Substance use	Frequency or quantity of other substance use
Frequency of primary substance use	Substance use	Frequency or quantity of primary substance use
Functioning issues already addressed	Functioning	Functioning issues already being addressed by another treatment provider
Gender	Demographics	Gender identification
Highest education	Demographics	Highest level of education completed
Homelessness	Demographics	Homeless or at risk of eviction
Hospitalisations due to mental health	Mental health	Recent hospitalisations due to mental health concern
Hospitalisations due to physical health	Physical health	Recent hospitalisations due to physical health concern
Injecting drugs	Substance use	Currently injecting drugs
Lifetime hospitalisations due to physical health	Physical health	Lifetime hospitalisations due to physical health concern
Living with children	Demographics	If the person is living with any children
Living with others	Demographics	Living with other people using alcohol or other drugs
Mental health issues already addressed	Mental health	Mental health comorbidities already being addressed by another treatment provider
Mental health severity	Mental health	Severity of mental health concerns
Nature CJS involvement	Demographics	Nature of involvement with criminal justice system
OAT	Substance use	Currently engaging with opioid agonist treatment
Other substances	Substance use	Other substances of concern
Overdose risk	Substance use	Overdose risk
Past AOD treatment	Substance use	Types/outcomes of past alcohol or other drug treatment
Physical health issues already addressed	Physical health	Physical health comorbidities already being addressed by another treatment provider
Preferred language	Demographics	Preferred language (other than English)
Pregnant or breastfeeding	Physical health	Person is currently pregnant or breastfeeding
Prescribed medications for mental health	Mental health	Currently using any prescribed medications for mental health concern
Prescribed medications for physical health	Physical health	Currently using any prescribed medications for physical health concern
Primary substance	Substance use	Primary substance of concern
Quality of life	Functioning	The person's quality of life
Refugee status	Demographics	Refugee Status
Relationship status	Demographics	Relationship status
Sex	Demographics	Sex assigned at birth
Sexual orientation	Demographics	Sexual orientation
Sharing equipment	Substance use	Currently sharing injecting equipment
Substance use issues already addressed	Substance use	Substance use issues already being addressed by another treatment provider
SUD severity	Substance use	Severity of person's substance use disorder
Suicide/self‐harm	Mental health	Suicide or self‐harm risk
Transport	Demographics	Access to transport
Youth specific—abuse/neglect	Youth/adolescent‐specific	Experience of abuse or neglect
Youth specific—child protection	Youth/adolescent‐specific	Child protection concerns
Youth specific—CJS involvement	Youth/adolescent‐specific	involvement with criminal justice system
Youth specific—disengagement	Youth/adolescent‐specific	Disengagement with education or training
Youth specific—nature CJS involvement	Youth/adolescent‐specific	Nature of involvement with criminal justice system
Youth specific—outside parental home	Youth/adolescent‐specific	Living outside parental home
Youth specific—parent AOD	Youth/adolescent‐specific	Parent's substance use

### 2.5 Analyses

All analyses were conducted using SPSS Version 26. Variables were summarised using frequencies and valid percentages, as appropriate. Missing cases for individual variables are reported in table notes. The workforce characteristics of survey participants were examined according to metropolitan or non‐metropolitan location.

The pre‐determined list of variables indicating client characteristics and clinical profile was analysed using percentages and rank ordered according to percentage most frequently rated as either ‘very important’ or ‘essential’ in identifying treatment need. Comparisons were made between those who worked in metropolitan versus non‐metropolitan locations, and according to whether they were an allied health worker (referent category), medical professional, nurse and/or manager. Where participants endorsed ‘Other (specify)’, free text responses were subsequently re‐categorised where appropriate and/or summarised in text.

## 3 RESULTS

### 3.1 Workforce characteristics of the sample

The total sample (*N* = 188) was comprised of participants reporting diverse job roles (Table 2). On average, participants had worked in the AOD sector for 12 years (*M* = 11.61, SD = 9.6) and in their current job role for 6 years (*M* = 6.04, SD = 6.56). More than two‐thirds of participants worked in community/non‐residential/outpatient treatment settings, and the majority worked in the government sector (Table 2).

**TABLE 2:** Characteristics of survey participants.

	Total sample	A. Metropolitan	B. Regional/rural	A versus B	
	*N* = 188%	*n* = 115%	*n* = 73%	OR (95% CI)	
Setting					
Outpatient					
Community/non‐residential/outpatient	**71.2**	**65.2**	**83.6**	2.71** (1.31, 5.62)	
Outreach/home visit	4.7	5.2	4.1	0.80 (0.19, 3.22)	
Inpatient					
NGO residential/inpatient	3.7	3.5	4.1	1.19 (0.26, 5.47)	
Hospital and/or inpatient	**15.7**	**24.3**	**2.7**	0.09*** (0.02, 0.38)	
Other^a^	3.1	1.7	5.5	3.28 (0.58, 18.36)	
Current job role					
Medical practitioner^b^	12.0	13.9	9.6	0.66 (0.26, 1.68)	
Nurse^c^	36.6	38.2	35.6	0.89 (0.49, 1.64)	
Counsellor^d^	14.1	13.9	15.1	1.10 (0.48, 2.52)	
Case manager/AOD worker^e^	7.4	6.1	9.6	1.64 (0.55, 4.87)	
Other allied health professional^f^	16.8	19.1	13.7	0.67 (0.30, 1.51)	
Health service manager^g^	11.5	8.7	16.4	2.07 (0.84, 5.06)	
Sector^h^					
Government organisation^i^	86.6	87.0	85.9	1.02 (0.44, 1.99)	
Non‐government organisation	13.4	13.0	14.1	0.93 (0.44, 1.99)	
	**Mean (SD)**	**Mean (SD)**	**Mean (SD)**	***t***	***p*‐value**
No. years working in current role	6.04 (6.56)	5.79 (6.51)	6.67 (6.91)	‐0.882	0.379
No. years worked in AOD sector	11.61 (9.67)	11.10 (9.22)	12.86 (10.22)	‐1.238	0.217

*Note*: Bold indicates statistical significance: *p < 0.05, ** *p* < 0.01, *** *p* < 0.001.

Abbreviations: AOD, alcohol and other drugs; CI, confidence interval; NGO, non‐government organisation; OR, odds ratio.

^a^ Other includes Aboriginal medical service, custodial settings and other drug and alcohol settings not specified above.

^b^ Medical practitioner includes addiction medicine specialist, medical practitioner and resident medical officer.

^c^ Nurse includes clinical nurse specialist/consultant, drug and alcohol nurse, general registered nurse, mental health nurse, nurse practitioner and midwife.

^d^ Counsellor includes counsellor and ‘drug and alcohol counsellor’.

^e^ Case manager/AOD worker includes alcohol and other drug worker, community worker, detox worker, family support worker, peer support worker and clinical support officer.

^f^ Other allied health professional includes clinical psychologist, psychologist, social worker, youth worker and occupational therapist.

^g^ Health service manager includes health service manager and nursing unit manager.

^h^ *n* = 2 participants working in primary care settings excluded.

^i^ Government organisation includes local health district and specialty health network.

Compared to those working in metropolitan areas, participants who worked in regional/rural areas were significantly more likely to work in community/non‐residential/outpatient settings and were less likely to work in hospital and/or inpatient settings (Table 2). There were no other significant differences in workforce characteristics among those working in regional/rural versus metropolitan areas.

### 3.2 Participants' overall rankings of the importance of individual client variables

More than three‐quarters of the individual client variables included in the survey (43 out of 56 variables in total) were ranked by the majority (>50%) of participants as either ‘very important’ or ‘essential’ in identifying treatment need. The top 10 individual variables ranked as ‘very important’/‘essential’ by over 90% of participants were ‘pregnant or breastfeeding’, ‘suicide/self‐harm’, ‘overdose risk’, ‘mental health severity’, ‘dependent children’, ‘co‐existing mental health concerns’, ‘hospitalisations due to mental health’, ‘child protection’ (for youth/adolescent populations) and ‘disability’. The 10 variables most commonly ranked as ‘slightly important’/‘not at all important’ included ‘citizenship’, ‘sex’, ‘country of birth’, ‘highest education’, ‘sexual orientation’, ‘relationship status’, ‘gender’, ‘transport’, ‘employment’ and ‘refugee status’.

### 3.3 Participants' rankings of the importance of individual client variables within domains, according to remoteness and job role

**FIGURE A1:**
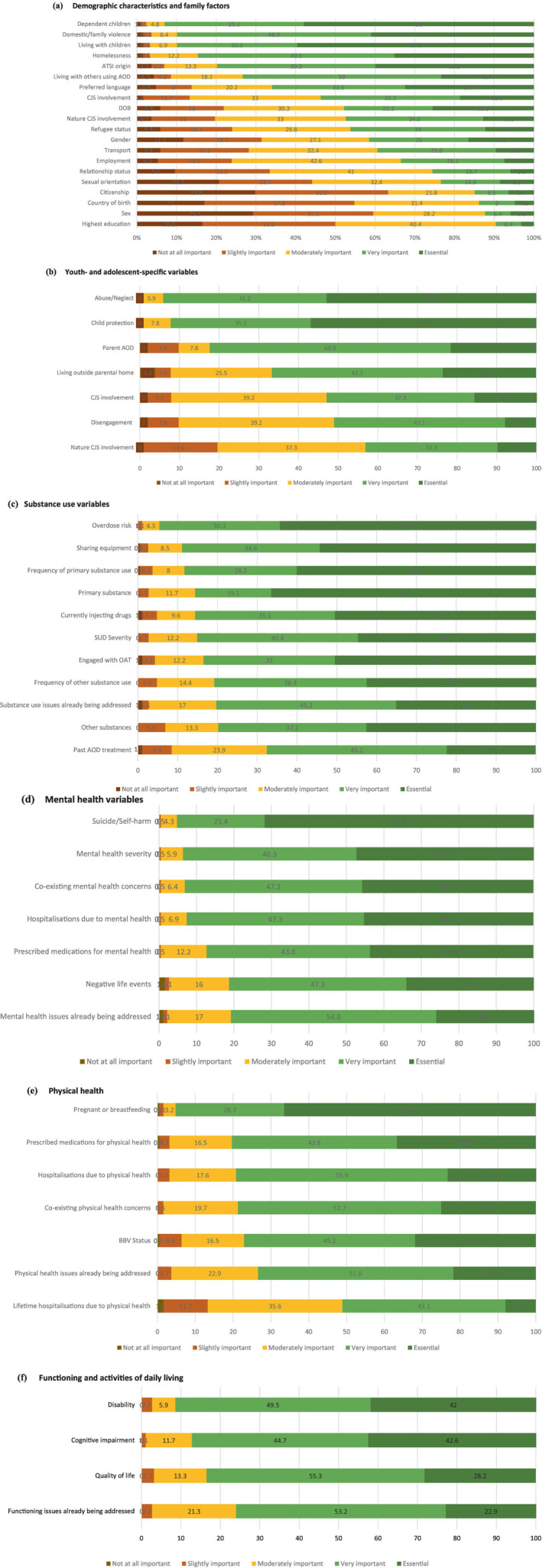
Participant ratings of how important client variables were identifying treatment need on a five‐point Likert scale (where 1 = ‘not at all important’, and 5 = ‘essential’). (a) Demographic characteristics and family factors. (b) Youth‐ and adolescent‐specific variables. (c) Substance use variables. (d) Mental health variables. (e) Physical health. (f) Functioning and activities of daily living.

A detailed breakdown of ratings on the original five‐point Likert scale of importance for each client variable is presented in Figure A1. The following reports the percentage of participants rating each variable as either ‘very important’ or ‘essential’.

#### 3.3.1 Demographics and family factors

Among the 20 potential variables describing demographics and family factors, the five most reported as ‘very important/essential’ were ‘dependent children’, ‘experience of domestic and family violence’, ‘living with children’, ‘homelessness’ and ‘Aboriginal and/or Torres Strait Islander origin’ (Table 3). Compared to metropolitan participants, rural/regional participants were less likely to rate ‘preferred language’, ‘nature of criminal justice system involvement’, ‘employment’ and ‘sexual orientation’ as ‘very important/essential’ (Table 3). Compared to allied health workers, managers were less likely to rate ‘homelessness’, ‘preferred language’ and ‘refugee status’ as ‘very important/essential’; and nurses were more likely to rate ‘living with children’ as ‘very important/essential’ (Table 3).

**TABLE 3:** Rankings of client variables as ‘very important’ or ‘essential’ in identifying treatment needs, by geographic location and job role.

Domain	Total sample	Geographic location			Job role						
		A. Metro	B. Regional/rural	A. versus B.	C. Allied health worker^a^	D. Medical professional^b^	E. Nurse^c^	F. Manager^d^	C. versus D.	C. versus E.	C. versus F.
	*N* = 188%	*n* = 115%	*n* = 73%	OR (95% CI)	*n* = 73%	*n* = 23%	*n* = 70%	*n* = 22%	OR (95% CI)	OR (95% CI)	OR (95% CI)
Demographics											
Dependent children	93.1	92.2	94.5	1.47 (0.43, 4.94)	90.4	95.7	94.3	95.5	2.33 (0.27, 20.03)	1.75 (0.49, 6.26)	2.23 (0.26, 19.16)
Domestic and family violence	89.9	90.4	89.0	0.86 (0.33, 2.25)	90.4	91.3	91.4	81.8	1.11 (0.22, 5.78)	1.12 (0.36, 3.55)	0.48 (0.13, 1.81)
Living with children	89.9	90.4	89.0	0.86 (0.33, 2.25)	**84.9**	82.6	**95.7**	95.5	0.84 (0.24, 2.96)	2.96* (1.06, 14.87)	3.73 (0.45, 30.61)
Homelessness	84.6	86.1	82.2	0.75 (0.34, 1.66)	**86.3**	87.0	88.6	**63.6**	1.06 (0.27, 4.23)	1.23 (0.46, 3.23)	0.28* (0.09, 0.83)
ATSI origin	79.8	78.3	82.2	1.28 (0.61, 2.70)	79.5	73.9	84.3	72.7	0.73 (0.25, 2.18)	1.39 (0.59, 3.27)	0.69 (0.23, 2.07)
Living with others using AOD	73.4	71.3	76.7	1.33 (0.67, 2.61)	75.3	60.9	80.0	59.1	0.51 (0.19, 1.37)	1.31 (0.59, 2.89)	0.47 (0.17, 1.29)
Preferred language	**66.0**	**72.2**	**56.2**	**0.49 (0.27, 0.92)**	**69.9**	69.6	67.1	**45.5**	0.99 (0.36, 2.73)	0.88 (0.44, 1.79)	0.36* (0.14, 0.95)
CJS involvement	53.7	57.4	47.9	0.68 (0.38, 1.23)	50.7	47.8	60.0	50.0	0.89 (0.35, 2.28)	1.46 (0.75, 2.83)	0.97 (0.38, 2.52)
Nature CJS involvement	**47.3**	**53.9**	**37.0**	**0.50 (0.28, 0.91)**	52.1	34.8	47.1	45.5	0.49 (0.19, 1.30)	0.82 (0.43, 1.58)	0.77 (0.30, 2.00)
DOB	47.1	47.0	49.3	1.10 (0.61, 1.98)	52.1	43.5	47.1	40.9	0.71 (0.23, 1.82)	0.82 (0.43, 1.58)	0.64 (0.24, 1.68)
Refugee status	46.3	46.1	46.6	1.02 (0.57, 1.84)	**53.4**	60.9	40.0	**27.3**	1.36 (0.52, 3.53)	0.58 (0.30, 1.13)	0.33* (0.12, 0.93)
Gender	41.5	45.2	35.6	0.67 (0.37, 1.23)	50.7	34.8	38.6	27.3	0.52 (0.20, 1.37)	0.51 (0.31, 1.19)	0.37 (0.13, 1.04)
Transport	39.4	33.9	47.9	1.80 (0.99, 3.27)	38.4	34.8	40.0	45.5	0.86 (0.32, 2.82)	1.07 (0.55, 2.10)	1.34 (0.51, 3.51)
Employment	**33.5**	**40.0**	**23.3**	**0.46 (0.24, 0.88)**	35.6	47.8	28.6	27.3	1.66 (0.64, 4.28)	0.72 (0.36, 1.47)	0.68 (0.24, 1.94)
Relationship status	25.5	28.7	20.5	0.64 (0.32, 1.29)	23.3	30.4	30.0	13.6	1.44 (0.51, 4.08)	1.41 (0.67, 2.98)	0.52 (0.14, 1.97)
Sexual orientation	**23.4**	**28.7**	**15.1**	**0.44 (0.21, 0.94)**	24.7	26.1	21.4	22.7	1.08 (0.37, 3.15)	0.83 (0.38, 1.82)	0.90 (0.29, 2.78)
Citizenship	14.9	17.4	11.0	0.56 (0.24, 1.41)	15.1	26.1	11.4	13.6	1.99 (0.64, 6.16)	0.73 (0.27, 1.93)	0.89 (0.23, 3.52)
Country of birth	13.8	11.3	17.8	1.70 (0.74, 3.91)	17.8	8.7	14.3	4.5	0.44 (0.91, 2.11)	0.77 (0.31, 1.89)	0.22 (0.23, 1.78)
Sex	12.2	11.3	13.7	1.25 (0.52, 3.01)	9.6	17.4	12.9	13.6	1.99 (0.53, 7.51)	1.39 (0.49, 3.96)	1.49 (0.35, 6.32)
Highest education	9.6	9.6	9.6	1.00 (0.37, 2.72)	6.8	17.4	10.0	9.1	2.86 (0.70, 11.72)	1.51 (0.46, 5.01)	1.36 (0.25, 7.55)
Substance use											
Overdose risk	94.7	94.8	94.5	0.95 (0.26, 3.49)	70 (95.9)	20 (87.0)	66 (94.3)	22 (100.0)	0.29 (0.05, 1.53)	0.71 (0.15, 3.28)	‐
Sharing equipment	88.8	87.8	90.4	1.31 (0.50, 3.41)	**84.9**	73.9	**97.1**	90.0	0.50 (0.16, 1.56)	6.03* (1.29, 28.29)	1.77 (0.36, 8.69)
Frequency of primary substance use	88.3	85.2	93.2	2.36 (0.83, 6.70)	83.6	87.0	91.4	95.5	1.31 (0.34, 5.12)	2.10 (0.74, 5.94)	4.12 (0.51, 33.72)
Currently injecting drugs	85.6	82.6	90.4	1.99 (0.79, 4.96)	80.0	91.3	90.0	81.8	2.49 (0.52, 11.89)	2.14 (0.81, 5.66)	1.07 (0.31, 3.65)
Primary substance	85.6	84.3	87.7	1.32 (0.56, 3.12)	78.1	100.0	88.6	86.4	‐	2.18 (0.87, 5.47)	1.78 (0.47, 6.78)
SUD severity	85.1	84.3	86.3	1.17 (0.51, 2.70)	79.5	91.3	90.0	81.8	2.72 (0.57, 12.89)	2.33 (0.89, 6.11)	1.16 (0.34, 3.95)
Engaged with OAT	83.5	87.0	78.1	0.53 (0.25, 1.16)	**74.0**	91.3	**91.4**	81.8	3.69 (0.79, 17.26)	3.75** (1.40, 10.07)	1.58 (0.48, 5.27)
Frequency of other substance use	80.9	80.9	80.8	1.00 (0.47, 2.10)	**71.2**	78.3	**91.4**	81.8	1.45 (0.48, 4.42)	4.31** (1.62, 11.46)	1.82 (0.55, 6.01)
Substance use issues already being addressed	80.3	80.9	79.5	0.92 (0.44, 1.91)	76.7	91.3	80.0	81.8	3.19 (0.68, 15.00)	1.21 (0.55, 2.70)	1.37 (0.41, 4,59)
Other substances	79.8	80.0	79.5	0.97 (0.47, 2.00)	74.0	91.3	84.3	72.7	3.69 (0.79, 17.26)	1.89 (0.82, 4.33)	0.94 (0.32, 2.75)
Past AOD treatment	67.6	68.7	65.8	0.88 (0.47, 1.63)	61.6	82.6	68.6	68.2	2.96 (0.91, 9.59)	1.36 (0.68, 2.71)	1.33 (0.48, 3.67)
Physical health											
Pregnant or breastfeeding	95.2	94.8	95.9	1.28 (0.31, 5.30)	91.8	100.0	95.7	100.0	‐	2.00 (0.48, 8.33)	‐
Prescribed medications for physical health	80.3	77.4	84.9	1.65 (0.76, 3.58)	74.0	91.3	84.3	77.3	3.69 (0.79, 17.26)	1.89 (0.82, 4.33)	1.20 (0.39, 3.69)
Hospitalisations due to physical health	79.3	80.0	78.1	0.89 (0.43, 1.83)	**72.6**	**95.7**	80.0	81.8	8.30* (1.05, 65.72)	1.51 (0.68, 3.29)	1.70 (0.51, 5.63)
Co‐existing physical health concerns	78.7	80.0	76.7	0.82 (0.41, 1.67)	71.2	87.0	82.9	81.8	2.69 (0.72, 10.03)	1.95 (0.88, 4.35)	1.82 (0.55, 6.01)
BBV status	77.1	77.4	76.7	0.96 (0.48, 1.93)	**64.4**	87.0	**88.6**	72.7	3.69 (1.00, 13.60)	4.29** (1.78, 10.32)	1.48 (0.51, 4.23)
Physical health issues already being addressed	73.4	73.9	72.6	0.94 (0.48, 1.81)	72.6	82.6	72.9	68.2	1.79 (0.54, 5.12)	1.01 (0.49, 2.12)	0.81 (0.29, 1.27)
Lifetime hospitalisations due to physical health	51.1	53.0	47.9	0.82 (0.45, 1.47)	57.5	56.5	44.3	45.5	0.96 (0.37, 2.47)	0.59 (0.30, 1.14)	0.62 (0.23, 1.61)
Mental health											
Suicide/self‐harm	95.2	93.0	98.6	5.38 (0.66, 43.97)	94.5	95.7	94.3	100.0	1.28 (0.14, 12.02)	0.96 (0.23, 3.98)	‐
Mental health severity	93.6	91.)	97.3	3.38 (0.72, 15.89)	94.5	100.0	90.0	95.5	‐	0.52 (0.15, 1.87)	1.22 (0.13, 11.49)
Co‐existing mental health concerns	93.1	91.3	95.9	2.22 (0.59, 8.36)	93.2	87.0	92.9	100.0	0.49 (0.12, 2.23)	0.96 (0.26, 3.46)	‐
Hospitalisations due to mental health	92.6	89.6	97.3	4.14 (0.90, 19.05)	93.2	91.3	90.0	100.0	0.77 (0.14, 4.27)	0.66 (0.20, 2.19)	‐
Prescribed medications for mental health	87.2	87.0	87.7	1.07 (0.44, 2.58)	86.3	91.3	87.1	86.4	1.67 (0.34, 8.23)	1.08 (0.41, 2.83)	1.01 (0.25, 4.03)
Adverse life events	81.4	81.7	80.8	0.94 (0.44, 1.99)	83.6	82.6	77.1	86.4	0.93 (0.27, 3.24)	0.66 (0.29, 1.53)	1.25 (0.32, 4.88)
Mental health issues already being addressed	80.9	79.1	83.6	1.34 (0.62, 2.88)	79.5	87.0	78.6	86.4	1.72 (0.45, 6.58)	0.95 (0.42, 2.12)	1.64 (0.43, 6.28)
Functioning											
Disability	91.5	93.0	89.0	0.61 (0.22, 1.70)	91.8	95.7	90.0	90.9	1.9 (0.23, 17.27)	0.81 (0.26, 2.53)	0.90 (0.17, 4.79)
Cognitive impairment	87.2	87.0	87.7	1.07 (0.44, 2.58)	83.6	95.7	84.3	100.0	4.33 (0.53, 35.25)	1.05 (0.43, 2.58)	‐
Quality of life	83.5	81.7	86.3	1.41 (0.62, 3.19)	90.4	82.6	78.6	77.3	0.50 (0.13, 1.91)	0.39 (0.15, 1.02)	0.36 (0.10, 1.28)
Functioning issues already being addressed	76.1	77.4	74.0	0.83 (0.42, 1.64)	74.0	78.3	77.1	77.3	1.27 (0.41, 3.88)	1.19 (0.55, 2.55)	1.20 (0.39, 3.69)
	***N* = 51%**	***n* = 25%**	***n* = 26%**	**OR (95% CI)**	***n* = 73%**	***n* = 23%**	***n* = 70%**	***n* = 22%**	**OR (95% CI)**	**OR (95% CI)**	**OR (95% CI)**
Youth and adolescent‐specific											
Abuse/neglect	94.1	92.0	96.2	2.17 (0.19, 25.61)	96.0	100.0	92.3	85.7	‐	0.50 (0.03, 8.71)	0.25 (0.01, 4.60)
Child protection	92.2	92.0	92.3	1.04 (0.14, 8.04)	92.0	100.0	92.3	85.7	‐	1.04 (0.09, 12.71)	0.52 (0.04, 6.77)
Parent AOD	82.4	76.0	88.5	2.42 (0.53, 11.00)	88.0	83.3	69.2	85.7	0.682 (0.06, 8.00)	0.31 (0.06, 1.66)	0.82 (0.07, 9.36)
Living outside parental home	66.7	68.0	65.4	0.89 (0.28, 2.85)	68.0	100.0	53.8	57.1	‐	0.55 (0.14, 2.18)	0.63 (0.13, 3.49)
CJS involvement	52.9	52.0	53.8	1.08 (0.36, 3.24)	56.0	66.7	46.2	42.9	1.57 (0.24, 10.22)	0.67 (0.18, 2.59)	0.50 (0.09, 2.73)
Disengagement	51.0	60.0	42.3	0.49 (0.16, 1.49)	**60.0**	83.3	**23.1**	42.9	3.33 (0.34, 32.60)	0.20* (0.04, 0.91)	0.59 (0.12, 3.20)
Nature CJS involvement	43.1	52.0	34.6	0.49 (0.16, 1.51)	56.0	16.7	38.5	28.6	0.16 (0.02, 1.55)	0.49 (0.13, 1.93)	0.31 (0.05, 1.94)

*Note*: Bold indicates statistical significance: *p < 0.05, ** *p* < 0.01, *** *p* < 0.001.

Abbreviations: AOD, alcohol and other drugs; ATSI, Aboriginal or Torres Strait Islander origin; BBV, blood borne virus; CI, confidence interval; CJS, criminal justice system; DOB, date of birth; OAT, opioid agonist treatment; OR, odds ratio; SUD, substance use disorder.

^a^ Allied health worker includes counsellor, alcohol and other drug counsellor, alcohol and other drug worker, community worker, detox worker, family support worker, peer support worker, clinical support officer, clinical psychologist, psychologist, social worker, youth worker and occupational therapist.

^b^ Medical professional includes addiction medicine specialist, medical practitioner and resident medical officer.

^c^ Nurse includes clinical nurse specialist/consultant, alcohol and other drug nurse, general registered nurse, mental health nurse, nurse practitioner and midwife.

^d^ Manager includes health service manager and nursing unit manager.

#### 3.3.2 Substance use

All of the 11 potential substance use variables were rated as ‘very important/essential’ by the majority (over two‐thirds) of participants. The five most reported as ‘very important/essential’ were ‘overdose risk’, ‘sharing equipment’, ‘frequency of primary substance use’, ‘currently injecting drugs’, and ‘primary substance of concern’. There were no differences in importance ratings among regional/rural versus metropolitan participants (Table 3). Compared to allied health workers, nurses were more likely to rate ‘sharing equipment’, ‘engaged with opioid agonist treatment’ and ‘frequency of other substance use’ as ‘very important/essential’ (Table 3).

#### 3.3.3 Physical health

Six out of seven physical health variables were rated as ‘very important/essential’ by the majority (>70%) of participants. The five most rated as ‘very important/essential’ were ‘pregnant or breastfeeding’, ‘prescribed medications for physical health’, ‘hospitalisations due to physical health’, ‘co‐existing physical health concerns’ and ‘blood‐borne virus status’. No difference in rankings were identified between metropolitan and non‐metropolitan participants (Table 3). Compared to allied health workers, doctors were more likely to rate ‘hospitalisations due to physical health’ as ‘very important/essential’ and nurses were more likely to rate ‘blood‐borne virus status’ as ‘very important/essential’ (Table 3).

#### 3.3.4 Mental health

All of the seven mental health variables were rated as ‘very important/essential’ by the vast majority (>80%) of participants. The five most rated as ‘very important/essential’ were ‘suicide/self‐harm’, ‘mental health severity’, ‘co‐existing mental health concerns’, ‘hospitalisations due to mental health’ and ‘prescribed medications for mental health’. No difference in rankings were identified between metropolitan and non‐metropolitan participants or according to job role (Table 3).

#### 3.3.5 Functioning and activities of daily living

All four potential functioning/activities of daily living variables were rated as ‘very important/essential’ by more than three‐quarters of participants. Their ranking in order of importance was ‘disability’, ‘cognitive impairment’, ‘quality of life’ and ‘functioning issues already being addressed by another provider’. No difference in rankings were identified between metropolitan and non‐metropolitan participants or according to job role (Table 3).

#### 3.3.6 Youth‐specific

Among the subset of participants who indicated working with youth and/or adolescent populations (*n* =51), four of seven youth and adolescent‐specific variables were ranked as ‘very important/essential’ by more than two‐thirds of participants. The five most rated as ‘very important/essential’ were ‘abuse/neglect’, ‘child protection’, ‘parental alcohol and other drug use’, ‘living outside of parental home’ and ‘criminal justice system involvement’. There were no differences in importance ratings among regional/rural versus metropolitan participants (Table 3). Compared to allied health workers, nurses were less likely to rate ‘disengagement from education’ as ‘very important/essential’ (Table 3).

### 3.4 Validated measures and screening tools used to assess treatment need

The measures most used by the total sample to assess treatment need, client complexity and inform client care at the beginning of a treatment episode were the ATOP [10], Kessler Psychological Distress Scale (K10) [6], the Client Complexity Rating Scale [14], the Severity of Dependence Scale [15] and the Alcohol and Drug Cognitive Enhancement Screening Tool [16] (Table 4). Regional/rural participants were more likely to use the K10 and the Alcohol and Drug Cognitive Enhancement Screening tool (Table 4). Compared to NGOs, government participants were more likely to report use of the ATOP and the Client Complexity Rating Scale, and were less likely to use the K10, the WHO‐8/EUROHIS QoL (an 8‐item index taken from the World Health Organization Quality of Life Instrument‐Abbreviated Version [17]), Severity of Dependence Scale and Primary Care Post‐Traumatic Stress Disorder Screener (Table 4).

**TABLE 4:** Percentage of participants reporting use of additional measures to assess treatment need, client complexity and inform client care at the beginning of a treatment episode, by geographic location and sector.

	Geographic location				Sector		
	Total sample	A. Metropolitan	B. Regional/rural	A. versus B	C. Government	D. Non‐government	C. versus D.
	*N* = 188%	*n* = 115%	*n* = 73%	OR (95% CI)	*n* = 161%	*n* = 25%	OR (95% CI)
Australian Treatment Outcome Profile	66.5	63.5	71.2	1.43 (0.76, 2.68)	**70.8**	**44.0**	0.32* (0.14, 0.77)
Kessler Psychological Distress Scale (K10)	30.3	**24.3**	**39.7**	2.05* (1.09, 3.86)	**24.2**	**64.0**	5.56*** (2.28, 13.58)
Client Complexity Rating Scale	27.7	30.4	23.3	0.69 (0.35, 1.36)	**32.3**	**0.0**	***
Substance Dependence Scale	22.9	22.6	23.3	1.04 (0.52, 2.09)	**19.3**	**44.0**	3.30* (1.37, 7.96)
Other^a^	19.7	20.0	19.2	0.95 (0.45, 1.99)	17.4	36.0	2.67 (1.07, 6.66)
Alcohol and Drug Cognitive Enhancement Screening Tool	17.0	**12.2**	**24.7**	2.36* (1.09, 5.11)	16.1	16.0	0.99 (0.31, 3.12)
HEEADSSS Assessments	10.6	8.7	13.7	1.67 (0.66, 4.23)	11.2	4.0	0.33 (0.04, 2.60)
WHO‐8/EUROHIS Quality of Life Scale	9.6	7.8	12.3	1.66 (0.63, 4.39)	**1.2**	**60.0**	119.25*** (23.88, 595.30)
Health of the Nations Outcomes Scales	6.9	4.3	11.0	2.71 (0.85, 8.63)	6.2	8.0	1.31 (0.27, 6.38)
Depression, Anxiety, Stress Scale (DASS‐21)	6.4	7.0	5.5	0.78 (0.23, 2.67)	5.6	12.0	2.30 (0.58, 9.16)
Strengths and Difficulties Questionnaire	5.9	3.5	9.6	2.94 (0.83, 10.44)	5.0	8.0	1.66 (0.33, 8.32)
Montreal Cognitive Assessment	4.3	3.5	5.5	1.61 (0.39, 6.64)	4.3	4.0	0.92 (0.12, 7.78)
Primary Care PTSD Screener (PC‐PTSD‐5)	4.3	2.6	6.8	2.75 (0.64, 11.85)	**1.2**	**24.0**	25.11*** (4.73, 133.30)
Risk of Cognitive Impairment Tool	3.7	1.7	6.8	4.15 (0.78, 22.01)	4.3	0.0	‐
DSM‐5 Self‐Rated Level 1 Cross‐Cutting Symptom Measure	3.2	3.5	2.7	0.78 (0.14, 4.38)	3.7	0.0	‐

*Note*: Bold indicates statistical significance: *p < 0.05, ** *p* < 0.01, *** *p* < 0.001.

Abbreviations: ADHD, attention‐deficit/hyperactivity disorder; CI, confidence interval; DSM‐5, Diagnostic and Statistical Manual of Mental Disorders, Fifth Edition; OR, odds ratio; PTSD, post‐traumatic stress disorder; WHO, World Health Organization.

^a^ Other includes Acquired Brain Injury screening, Adolescent Cannabis Check Up, Adverse Childhood Experiences Screener, Alcohol Use Disorders Identification Test, Brief Symptom Inventory, Borderline Symptom List (BSL‐23), Community Health and Outpatient Care Nursing Assessment, Clinical assessment, Clinical Opiate Withdrawal Scale, Drug and Alcohol Nursing Assessment, Drug Use Disorders Identification Test, Depression Anxiety Stress Scale—42 (DASS‐42), Domestic Violence Screen, Edinburgh Depression Scale, Edinburgh Postnatal Depression Scale, Fagerstrom Test for Nicotine Dependence, General Anxiety Disorder Screener (GAD‐7), General Health Questionnaire, general wellbeing survey, Harm Reduction Model of Care Assessment, NADA Suicide Screener, neuropsychological consult, Outcome Rating Scale, Outcome Star, Patient Experience Questionnaire, Patient Health Questionnaire (PHQ‐9), Problem Gambling Severity Index, Rowland Universal Dementia Assessment Scale, service specific cognitive assessment tool, Substance Use Recovery Evaluator, and Vanderbilt ADHD Assessment Scale.

### 3.5 Additional important variables not captured in the survey

Participants' responses to ‘Other (please specify)’ indicated there are a range of variables used by service providers in identifying treatment need that were not captured in the survey items/domains:

- Demographic: cultural identity (separate from country of birth or language spoken at home), religious beliefs and preferred pronouns.
- Substance use: withdrawal complications, drug interactions and use of substances in sexual activities. Service providers also believed that broader assessments of the impacts of substance use on clients were important in treatment planning, including prior experience of withdrawal, impacts of substance use on others, impacts on employment/job loss, impacts on housing, criminal charges arising from substance use, medical or psychiatric complications of substance use.
- Physical health: contraception, safe sex practices, sexually transmitted diseases (status and history), acute physical health issues, current health status, allergies, complications of opioid agonist treatment, history of brain injury/seizure/stroke, current and past accidents/injuries, chronic pain, diabetes, hypercholesterolemia, weight issues, hypertension, malnutrition, COVID and immunisation status, identification of other medical/physical health issues associated with substance use, recent blood test results, access to a regular GP, Medicare eligibility and private health insurance.
- Mental health: mental health diagnosis, marginalisation and intergenerational trauma, risk to others and issues relating to mental health medications.
- Functioning and activities of daily living: nature and severity of disability/impairment, impact of substance use on disability (and vice versa), ability of client to advocate for their needs, client's assessment of functioning and disability, how effectively disability/impairment is being managed, National Disability Insurance Scheme involvement, current supports in place, capacity to engage with multiple intake processes, disability support pension, transport options, computer and phone literacy.
- Youth‐ and adolescent‐specific: neurodevelopmental difficulties, presence of safe adults, available support systems/social support, engagement with other services, intimate relationships, sexual health and accommodation needs and issues.

Participants identified several other areas important for assessment, including clients' prior treatment experiences and preferences, clients' treatment goals and motivations, clients' own caring responsibilities (e.g., children, ageing parents, pets), access issues (e.g., client's residential location, travel/transport issues, access to a phone and internet), financial and legal issues (e.g., financial hardship, income source, social security, whether there is a guardian or power of attorney in place).

## 4 DISCUSSION

This study aimed to identify the demographic and clinical variables that service providers believe are most important for informing care planning and determining intensity of AOD treatment interventions. More than three‐quarters of the individual potential variables included in this study were perceived by the most participants as either ‘very important’ or ‘essential’ in identifying treatment need, with little differentiation in terms of relative importance. Among the total list of potential variables, the top 10 variables service providers believed were ‘very important/essential’ were variables relating to immediate risk of harm to the client (i.e., suicidality/self‐harm, overdose risk), priority populations (i.e., pregnancy or breastfeeding, disability), child protection concerns (i.e., child protection concerns among youth/adolescent populations, dependent children, abuse/neglect among youth/adolescent clients), and mental health comorbidity (i.e., co‐occurrence and severity of mental health concerns, history of hospitalisations due to mental health).

Although many of these variables are likely identified through comprehensive, service‐specific client assessments, some of these (e.g., suicidality/self‐harm, pregnancy/breastfeeding, disability or significant cognitive, sensory or physical impairment) are not currently captured routinely (or are captured only indirectly) in the most common routinely collected and reported data systems (Table A1). For example, in government organisations, suicidality/self‐harm is prompted for in clinical findings and will be reported through the NSW Non‐Admitted Patient Data Collection [9] in the future. Although not currently routinely reported, pregnancy/breastfeeding is being added to future revisions of the NSW MDS DATS [8]. There are some variables where there is good coverage in existing systems, including Aboriginal and Torres Strait Islander status, primary substance of concern, homelessness (or insecure accommodation) and recent injection of drugs. Although all NSW data systems can identify whether clients live with children, only the ATOP [10] identifies whether there are dependent children and the presence of domestic and family violence. None of the systems routinely record child protection concerns or co‐occurring mental health and/or physical health concerns (blood‐borne virus status, diagnoses, recent hospitalisations and medications) with any specificity. Only the NADABase COMS [11] collects data on prior overdose although current overdose risk may be ascertained by considering combinations of responses to ATOP items (such as quantity and frequency of depressant drug use, route of administration and recent injecting). Both the ATOP and NADABase COMS collect data on physical health, frequency of substance use, sharing of injecting equipment and quality of life.

Although fewer participants overall rated variables as ‘slightly important’/‘not at all important’, the 10 variables most rated as ‘slightly important’/‘not at all important’ related to cultural and linguistic diversity (citizenship, country of birth and refugee status), gender identity (sex at birth, gender), sexual orientation, relationship status, socioeconomic status (highest education, employment) and transport issues. Most of these variables are captured in the NSW MDS Alcohol and Other Drug Treatment Services, which is the dataset reported by all government‐funded treatment services (regardless of whether they are delivered from government or NGO sectors) used by funders and policymakers in monitoring trends in treatment episodes and planning services. Despite service providers ranking these variables as less important clinically, many of these variables are important social determinants of health [18]. They are also important to policy makers and funders monitoring treatment access among priority populations, including culturally and linguistically diverse communities, non‐heterosexual and gender‐diverse populations [19]. From a broader systems perspective, different data systems might aim to characterise clients, facilitate benchmarking of client outcomes, implement and/or support important care pathways for ‘complex’ AOD clients, facilitate continuous quality improvement projects, support internal management and planning systems, or inform funding models. No single set of variables is likely to meet all these needs, and the challenge is to ensure a range of variables are routinely captured that can meet the multiple needs and expectations of such data systems.

This study is subject to the usual limitations associated with cross‐sectional survey designs and convenience sampling methods. Information about numbers examined for eligibility, confirmed eligible and included in the study were not routinely collected, and we are unable to report these details. Asking service providers to comment retrospectively about the variables they think are most important to assess treatment need is likely subject to biases, including individual clinician heuristics and/or recent influential cases. In addition, service providers from the NGO sector services are under‐represented in this survey which limited statistical comparisons between those working in government and NGO sectors. Thirty‐nine percent of all treatment episodes in NSW are delivered by NGO [20]. In addition, this sector may provide services to clients with a range of complexities who do not access public services. Although not assessed in the current study, assessments of client complexity to inform care planning also need to consider client strengths and protective factors. Finally, results regarding the utility and importance of youth‐specific items should be interpreted with caution due to the small number of respondents working in youth specific services. Despite these limitations, this study provides important insights into what service providers believe guides their decision‐making regarding treatment difficulty/intensity. Further research should triangulate service providers' perceptions with the perceptions of people with lived and living experience of AOD services. Where casemix classification systems are being developed to inform treatment system planning and improvements (e.g., [21, 22, 23, 24, 25]), statistical modelling and health economic studies examining associations with resource utilisation will be needed.

This study represents a first step towards understanding whether data that are currently routinely collected meet clinician expectations regarding identifying treatment need. These data are important for informing service providers, funders and policymakers regarding the client factors believed to be most important in planning and funding of AOD treatment services. A better understanding of client characteristics that impact service delivery can be used to inform improvements in routine data collection in the sector, the evaluation of AOD treatment activities and funding models to help deliver services that are better tailored to the needs of clients. Although further validation is needed, many of the variables deemed most important by service providers in identifying treatment need and intensity are not captured in routine data collections; many of those variables deemed less important are routinely captured. It seems timely to review the data collection and reporting requirements to funders and policymakers to ensure that they are capturing the best variables to suit a range of different treatment‐ and system‐level purposes.

## AUTHOR CONTRIBUTIONS

Briony Larance: conceptualisation, funding acquisition, investigation, methodology, project administration, supervision, writing – original draft; Isabella Ingram: investigation, project administration, writing – reviewing and editing; Chloe Haynes: project administration, visualisation, writing – original draft, reviewing and editing; Lexi Buckfield: conceptualisation, investigation, methodology, writing – reviewing and editing; Choo Wee Melvin Goh: investigation, project administration, writing – reviewing and editing; Peter J. Kelly: conceptualisation, funding acquisition, investigation, methodology, writing – reviewing and editing.

## CONFLICT OF INTEREST STATEMENT

Lexi Buckfield is an employee of the Centre for Alcohol and Other Drugs, NSW Ministry of Health.

## Data Availability

Data available on request due to privacy/ethical restrictions.

## Appendix

**TABLE A1:** Mapping of equivalent measures of the current study's survey items against existing New South Wales alcohol and other drug data collections.

Survey items	Equivalent items in existing data collections (New South Wales)			
	New South Wales Minimum Data Set for Alcohol and other Drug Treatment Services [12]	New South Wales Non‐Admitted Patient Data Collection [13]	Australian Treatment Outcome Profile (ATOP) [10]	Client Outcomes Monitoring System (NADABase COMS) [11]
Demographic characteristics and family factors				
Date of birth (age)	Date of birth (Numeric response) DDMMYYYY	Client Date of Birth (EDW) (Numeric response) YYYYMMDD	Date of birth (Numeric response)	‐
Sex recorded at birth	Sex (Numeric response) 1—Male 2—Female 9—Not stated/inadequately described	Client Sex Code (EDW) (Numeric response) 1—Male 2—Female 3—Indeterminate 9—Unknown	Sex (Alphabetic response)	‐
Gender identity	Sex (Numeric response) 1—Male 2—Female 9—Not stated/inadequately described	Client Sex Code (EDW) (Numeric response) 1—Male 2—Female 3—Indeterminate 9—Unknown	‐	‐
Sexual orientation	‐	‐	‐	‐
Highest level of education completed	‐	‐	‐	‐
Access to transport (e.g., own car, public transport)	‐	‐	‐	‐
Work status/employment	Principal source of income (Numeric response) 01—Full‐time employment 02—Part‐time employment 03—Temporary benefit (e.g., unemployment) 04—Pension (e.g., aged, disability) 05—Student allowance 06—Dependent on others 07—Retirement fund 08—No income 98—Other 99—Not stated/not known/inadequately described	‐	‐	Principal source of income (taken from New South Wales Minimum Data Set) (Numeric response) 01—Full‐time employment 02—Part‐time employment 03—Temporary benefit (e.g., unemployment) 04—Pension (e.g., aged, disability) 05—Student allowance 06—Dependent on others 07—Retirement fund 08—No income 98—Other 99—Not stated/not known/inadequately described
Homelessness/risk of eviction	Usual accommodation (Numeric response) 01—Rented house or flat (public or private) 02—Privately owned house or flat 03—Boarding house 04—Hostel/supported accommodation services 05—Psychiatric hospital 06—Alcohol/other drug treatment residence 07—Shelter/refuge 08—Prison/detention centre 09—Caravan on a serviced site 10—No usual residence/homeless 98—Other 99—Not known	Accommodation Type Code (EDW) (Numeric response) 01.00—Private Residence, not further defined 01.01—Private Residence—Client or Family Owned 01.02—Private Residence—Private Rental 01.03—Private Residence—Public Rental 01.04—Private Residence—Independent Living Unit 03.00—Residential Aged Care Facility, not further defined 03.01—Residential Aged Care Facility—Low Level Care 03.02—Residential Aged Care Facility—High Level Care 04—Drug/Alcohol Community Residential 05—Mental Health Community Residential 06—Domestic supported living/group home 07—Boarding House 08—Homeless Persons' Shelter 09—Other Shelter/Refuge 10—Other Supported Accommodation 11—Prison/Detention Centre 12—Homeless/No usual residence 15—Brain Injury Transitional Living Unit 17—Indigenous Community/Settlement 18—Caravan/Mobile Home on a serviced site 19—Community residential, not elsewhere classified 20.01‐ Hospital—Acute 20.02—Hospital—Psychiatric 20.03—Hospital—Rehabilitation 20.99—Hospital—not elsewhere classified 24—Informal housing 97—Other Accommodation, not elsewhere classified 98—Declined to respond 99—Unknown/Not Applicable	Have you been homeless? Have you been at risk of eviction? (Record for the past 4 weeks; tick one for each question) Yes No Not answered	Usual accommodation (taken from New South Wales Minimum Data Set) (Numeric response) 01—Rented house or flat (public or private) 02—Privately owned house or flat 03—Boarding house 04—Hostel/supported accommodation services 05—Psychiatric hospital 06—Alcohol/other drug treatment residence 07—Shelter/refuge 08—Prison/detention centre 09—Caravan on a serviced site 10—No usual residence/homeless 98—Other 99—Not known
Aboriginal and/or Torres Strait Islander origin	Aboriginal or Torres Strait Islander origin (Numeric response) 1—Aboriginal but not Torres Strait Islander origin 2—Torres Strait Islander but not Aboriginal origin 3—Aboriginal and Torres Strait Islander origin 4—Neither Aboriginal nor Torres Strait Islander origin 9—Not stated	Client Indigenous Status Code (EDW) (Numeric response) 1—Aboriginal but not Torres Strait Islander 2—Torres Strait Islander but not Aboriginal 3—Both Aboriginal and Torres Strait Islander 4—Neither Aboriginal or Torres Strait Islander 8—Declined to Respond 9—unknown	‐	‐
Preferred language (other than English)	Preferred language (Numeric response recorded as four‐digit code)	Preferred Language (EDW) (Numeric response recorded as four‐digit code)	‐	‐
Country of birth	Country of birth (Numeric response recorded as a four‐digit code)	Client Country Of Birth Code (EDW) (Numeric response recorded as a four‐digit code)	‐	‐
Citizenship status	‐	‐	‐	‐
Identifying if the person is a refugee	‐	‐	‐	‐
Relationship status	‐	‐	‐	‐
If the person if responsible for any dependents (children)	‐	‐	Have you, at any time in the past 4 weeks, been a primary caregiver for or living with any child/children (i) under 5 yo? (ii) 5–15 yo? (Tick one for each part of question) Yes No Not answered	‐
If the person is living with children	Living arrangement (Numeric response) 01—Alone 02—Spouse/partner 03—Single parent with child(ren) 04—Spouse/partner and child(ren) 05—Parent(s) 06—Other relative(s) 07—Friend(s) 08—Friend(s)/ parent(s)/ relative(s) and child(ren) 98—Other 99—Not known/not stated/inadequately described	Usual living arrangements (Numeric response) 01—Alone 02—Spouse/partner only 05—Spouse/partner and child(ren) 06—Parent(s) only 07—Other related adult(s) only 10—Child(ren) only 11—Child(ren) and parent(s) 12—Child(ren) and other relatives 13—Child(ren) and unrelated adults 14—Child(ren), relatives and unrelated adults 15—Unrelated adults only 16—Unrelated adults and their child(ren) 90—Client declined to respond 98—Other living arrangement, nec 99—0—Not known/not stated/inadequately described	Have you, at any time in the past 4 weeks, been a primary caregiver for or living with any child/children (i) under 5 yo? (ii) 5–15 yo? (Tick one for each part of question) Yes No Not answered	Living arrangement (taken from New South Wales Minimum Data Set) (Numeric response) 01—Alone 02—Spouse/partner 03—Single parent with child(ren) 04—Spouse/partner and child(ren) 05—Parent(s) 06—Other relative(s) 07—Friend(s) 08—Friend(s)/ parent(s)/ relative(s) and child(ren) 98—Other 99—Not known/not stated/inadequately described
If the person is living with others who are currently using alcohol or other drugs	‐	‐	‐	‐
Experience of domestic or family violence	‐	‐	Has anyone been violent (incl. domestic violence) towards you? Have you been violent (incl. domestic violence) towards someone? (Record for the past 4 weeks; tick one for each question) Yes No Not answered	‐
Involvement with the criminal justice system	‐	‐	Have you been arrested? (Record for the past 4 weeks; tick one) Yes No Not answered	2 Brief Treatment Outcome Measure (BTOM) items on crime (Two questions ask the number of times the client has been arrested in the last 3 months and how many arrests were for offences committed in the last 3 months)
Identifying nature of involvement with the criminal justice system	‐	‐	‐	‐
Youth specific items				
Parental substance use	‐	‐	‐	‐
Living outside parental home	‐	‐	‐	‐
Child protection concerns	‐	‐	‐	‐
Abuse or neglect	‐	‐	‐	‐
Disengagement with education/training	‐	‐	Record days worked and at college, school or vocational training for the past 4 weeks (Numeric responses summed to provide total 0–28) A—Days paid work (incl. all paid work; not voluntary work) B—Days at school, tertiary education, vocational training	‐
Involvement with Juvenile Justice	‐	‐	‐	‐
Identifying nature of involvement with the criminal justice system	‐	‐	‐	‐
Substance use				
The person's primary substance of concern	Principal drug of concern (Numeric response) 0000—Inadequately described 0001—Not Stated 0005—Pharmaceutical opioids, nfd 0006—Psychostimulants, nfd 0009—Gambling 1202—Heroin 1305—Methadone 2101—Alcohol 2400—Benzodiazepines, nfd 3100—Amphetamines, nfd 3405—MDMA/Ecstasy 3901—Caffeine 3903—Cocaine 3906—Nicotine 7100—Cannabinoids and related Drugs, nfd Other substance (specify the ASCDC four‐digit code)	‐	‐	‐
The quantity/frequency the person uses their primary substance of concern	‐	‐	Record number of days used in each of the past 4 weeks (Numeric response to indicate typical quantity; Numeric responses to indicate number of days; summed to provided total 0–28) A—Alcohol B—Cannabis C—Amphetamine type substances (ice, MDMA etc.) D—Benzodiazepines (prescribed and illicit) E—Heroin F—Other opioids (not prescribed methadone/buprenorphine) G—Cocaine H(i)—Other substance H(ii)—Other substance I—Tobacco	4‐items from Brief Treatment Outcome Measure (BTOM) and Australian Alcohol Treatment Outcome Measure (Numeric responses) Number of days used for illicit drugs and benzodiazepines (0–28) Number of days used (0–28) and quantity used for alcohol and tobacco (number of days the person drank alcohol and average number of drinks per day, and number of days of heavier drinking than usual and average number of drinks on those days)
The person's other substance(s) of concern	Other drugs of concern/gambling (Numeric response) 0000—Inadequately described 0001—Not stated 0003—No other drugs of concern 0005—Opioid analgesics, nfd 0006—Psychostimulants, nfd 0009—Gambling 1202—Heroin 1305—Methadone 2101—Alcohol 2400—Benzodiazepines, nfd 3100—Amphetamines, nfd 3405—MDMA/Ecstasy 3901—Caffeine 3903—Cocaine 3906—Nicotine 7100—Cannabinoids and related Drugs, nfd Other substance (specify the ASCDC four‐digit code)	‐	‐	‐
The quantity/frequency the person uses their other substance(s) of concern	‐	‐	Record number of days used in each of the past 4 weeks (Numeric response to indicate number of days and typical quantity) A—Alcohol B—Cannabis C—Amphetamine type substances (ice, MDMA etc.) D—Benzodiazepines (prescribed and illicit) E—Heroin F—Other opioids (not prescribed methadone/buprenorphine) G—Cocaine H(i)—Other substance H(ii)—Other substance I—Tobacco	4‐items from Brief Treatment Outcome Measure (BTOM) and Australian Alcohol Treatment Outcome Measure (Numeric responses) Number of days used for illicit drugs and benzodiazepines (0–28) Number of days used (0‐28) and quantity used for alcohol and tobacco (number of days the person drank alcohol and average number of drinks per day, and number of days of heavier drinking than usual and average number of drinks on those days)
If the person is currently injecting drugs	Injecting drug use (Numeric response) 0—Not collected 1—Last injected within the previous 3 months 2—Last injected more than 3 months ago but less than 12 months ago 3—Last injected 12 months ago or more 4—Never injected 9—Not stated/inadequately described	‐	Record number of days client injected drugs in the past 4 weeks (Numeric responses) (If no, enter zero)	When did you last inject/hit up any drug? (Taken from Brief Treatment Outcome Measure) (Numeric response) 1—Last injected within the previous 3‐months 2—Last injected more than 3‐months ago 3—Last injected 12‐months ago 4—Never injected 5—Not stated/inadequately described
If the person shares injecting drug use equipment	‐	‐	Inject with equipment used by someone else? (Tick one) Yes No Not answered	How many times in the last 3 months did you use a needle and syringe after someone else had already used it (including your sex partner and even if it was cleaned)? (Taken from Brief Treatment Outcome Measure) (Numeric response) In the last 3 months did you share any spoons, filters, water, tourniquets, drug solution/mix, or swabs with anyone else? (Taken from Brief Treatment Outcome Measure) (Numeric response) 0—No 1—Yes
If the person is engaged with opioid agonist treatment (current)	Main service provided (Numeric response) 10—Counselling 20—Withdrawal management (detoxification) 30—Rehabilitation activities 40—Maintenance pharmacotherapy (Opioid) 48—Maintenance pharmacotherapy (non‐opioid) 50—Consultation activities 60—Support and case management only 70—Involuntary Drug and Alcohol Treatment 91—Assessment only 92—Information and education only 98—Other	‐	‐	‐
Overdose risk	‐	‐	Individual ATOP items regarding frequency and quantity of depressant drug use, route of administration, and recent injecting can be used together to give an indication of overdose risk.	How many times have you overdosed from any drug in the last 3 months? (Taken from Brief Treatment Outcome Measure) (Numeric response)
The severity of the person's substance use disorder	‐	‐	‐	Severity of Dependence Scale (Numeric response) Five‐item screening measure (range 0–15)
The type and outcomes of previous AOD treatment	Episode identifier (Integer) Used to identify a service episode; Establishment identifier (Alphanumeric agency code)—for the agency that provided the service N—State Identifier (State Code) N—Establishment Sector (Numeric Code): 1—Public 2—Private 3—Non‐government (funded) 4—Non‐government (not funded) A—Region Code (LHD) NNN—Establishment Number (Unique Agency Number within NSW) Reason for cessation of service episode (Numeric response) 01—Service completed 02—Transferred/referred to another service 03—Left without notice 04—Left against advice 05—Left Involuntarily (non‐compliance) 06—Moved out of area 07—Sanctioned by drug court/court diversion program 08—Imprisoned, other than drug court sanction 09—Released from prison 10—Died 98—Other 99—Not stated/inadequately described Referral to another service (Numeric response) 03—General practitioner 04—Medical officer/specialist 05—Psychiatric hospital 06—Other hospital 07—Residential community mental health care unit 08—Residential alcohol and other drug treatment agency 09—Other residential community care unit 10—Education institution 11—Non‐residential community mental health centre 12—Non‐residential alcohol and other drug treatment agency 13—Non‐residential community health centre 14—Other non‐health service agency 18—Workplace (EAP) 19—Family and child protection service 97—No referral 98—Other 99—Not stated/inadequately described	‐	‐	‐
If the person's substance use issues are already being addressed by another service provider	‐	‐	‐	‐
Physical health				
If the person has any co‐existing physical health concerns	‐	‐	Client's rating of physical health status (extent of physical symptoms and bothered by illness) (Numeric response) A 0–10 scale where 0 is poor and 10 is good	‐
If the person has had any recent hospitalisations for a physical health concern	‐	‐	‐	‐
The number of lifetime hospitalisations related to the target condition	‐	‐	‐	‐
If the person is currently using any prescribed medications for a physical health concern	‐	‐	‐	‐
The person's blood‐borne virus status (hepatitis C, HIV)	‐	‐	‐	‐
If the person is currently pregnant or breastfeeding	‐	‐	‐	‐
If the person's physical health co‐morbidities are already being addressed by another service provider	‐	‐	‐	‐
Mental health	‐	‐	‐	‐
If the person has any co‐existing mental health concerns	‐	‐	‐	‐
If the person has had any recent hospitalisations for a mental health concern	‐	‐	‐	‐
If the person is currently using any prescribed medications for a mental health concern	‐	‐	‐	‐
The severity of the person's mental health concerns			Client's rating of psychological health status (Numeric response) A 0–10 scale where 0 is poor and 10 is good	Kessler 10+ scale (K10+) (Numeric response) Measure of psychological distress. Each item ranges from 1—None of the time to 5—All of the time. Scores are summed across 10 items to produce a total ranging from 10 to 50.
Experience or risk of suicide or self‐harm	‐	‐	‐	‐
Exposure to negative life events (e.g., adverse childhood experiences, trauma)	‐	‐	‐	‐
The person's mental health concerns are already being addressed by another service	‐	‐	‐	‐
Functioning items	‐	‐	‐	‐
If the person has a disability (e.g., vision impairment, deaf or hard of hearing, intellectual disability, psychosocial disability, acquired brain injury, autism spectrum disorder, physical disability)	‐	‐	‐	‐
Cognitive impairment	‐	‐	‐	‐
A person's quality of life			Client's rating of overall quality of life (Numeric response) A 0–10 scale where 0 is poor and 10 is good	The World Health Organization Quality of Life eight questions (WHO QoL‐8, also known as the EUROHIS QoL‐8) (Numeric response)
If the person's disability/ies were already being addressed by another service	‐	‐	‐	‐

*Note*: All items in the survey were rated on a scale of 1—not at all important to 5—essential in terms of their importance in informing client care.

Abbreviations: AOD, alcohol and other drug; LHD, local health districts; MDS, minimum data set; NSW, New South Wales.
